# An evidence-informed policymaking (EIPM) competency profile for the Brazilian Health System developed through consensus: process and outcomes

**DOI:** 10.1186/s12961-023-01052-z

**Published:** 2023-10-12

**Authors:** Jorge Otávio Maia Barreto, Davi Mamblona Marques Romão, Cecilia Setti, Maria Lúcia Teixeira Machado, Rachel Riera, Romeu Gomes, Daienne Amaral Machado, João Abreu, Keitty Regina Cordeiro de Andrade, Laura dos Santos Boeira, Letícia Pozza, Nathan Mendes Souza, Patrícia Logullo, Roberta Borges Silva, Sandra Maria do Valle Leone de Oliveira, Sara Emanuela de Carvalho Mota, Tamille Sales Dias, Tereza Setsuko Toma, Silvio Fernandes da Silva

**Affiliations:** 1 Hospital Sírio-Libanês, São Paulo, Brazil; 2 Fiocruz (Oswaldo Cruz Foundation), Brasília, Brazil; 3 Veredas Institute, São Paulo, Brazil; 4 UFSCar (Federal University of São Carlos), São Paulo, Brazil; 5 Unifesp (Federal University of São Paulo), São Paulo, Brazil; 6 IFF/Fiocruz (National Institute of Women, Children and Adolescents Health Fernandes Figueira), Rio de Janeiro, Brazil; 7 Codeplan (Federal District Planning Company), Brasília, Brazil; 8 Impulso Gov, São Paulo, Brazil; 9 Ministry of Health, Brasília, Brazil; 10 ODD Studio, Barcelona, Spain; 11 UFMG (Federal University of Minas Gerais), Belo Horizonte, Brazil; 12 Centre for Statistics in Medicine (CSM), Nuffield Department of Orthopaedics, Rheumatology and Musculoskeletal Diseases (NDORMS), and UK EQUATOR Centre, University of Oxford, Oxford, United Kingdom; 13 Fiocruz (Oswaldo Cruz Foundation), Campo Grande, Brazil; 14 Brazilian National School of Public Administration, Brasília, Brazil; 15 Health Institute, São Paulo State Health Secretariat, São Paulo, Brazil

**Keywords:** Evidence-informed policymaking, Evidence-informed decision-making, Knowledge translation, Competency profile, Knowledge, Skills and attitudes

## Abstract

**Background:**

Evidence-informed policymaking (EIPM) requires a set of individual and organizational knowledge, skills and attitudes that should be articulated with background factors and needs. In this regard, the development of an EIPM competency profile is important to support the diagnosis, planning and implementation of EIPM.

**Purpose:**

To present the process and outcomes of the development of an EIPM competency profile by an expert committee, to be applied in different contexts of the Brazilian Health System.

**Methods:**

A committee of experts in EIPM shared different views, experiences and opinions to develop an EIPM competency profile for Brazil. In six consensus workshops mediated by facilitators, the committee defined from macro problems to key actions and performances essential for the competency profile. The development steps consisted of: (1) Constitution of the committee, including researchers, professionals with practical experience, managers, and educators; (2) Development of a rapid review on EIPM competency profiles; (3) Agreement on commitments and responsibilities in the processes; (4) Identification and definition of macro problems relating to the scope of the competency profile; and (5) Outlining of general and specific capacities, to be incorporated into the competency profile, categorized by key actions.

**Results:**

The development of the EIPM competency profile was guided by the following macro problems: (1) lack of systematic and transparent decision-making processes in health policy management; (2) underdeveloped institutional capacity for knowledge management and translation; and (3) incipient use of scientific evidence in the formulation and implementation of health policies. A general framework of key actions and performances of the EIPM Competency Profile for Brazil was developed, including 42 specific and general key actions distributed by area of activity (Health Management, Scientific Research, Civil Society, Knowledge Translation, and Cross-sectional areas).

**Conclusions:**

The competency profile presented in this article can be used in different contexts as a key tool for the institutionalization of EIPM.

**Supplementary Information:**

The online version contains supplementary material available at 10.1186/s12961-023-01052-z.

## Background

Evidence-informed policymaking (EIPM) is the result of systematic and transparent processes to use different types of evidence, including scientific evidence, to inform decision-making in the formulation and implementation of policies and systems in health and other social areas. It takes place through effective communication and collaboration between producers and users of scientific knowledge, including a range of interested social groups [1, 2]. For evidence-informed decision-making (EIDM) to be part of the institutional processes of organizations responsible for health policies and systems, evidence should be used in conjunction with contextual factors, including public opinion, equity, feasibility of implementation, affordability, sustainability, and acceptability to stakeholders [3].

Knowledge translation—a foundation of EIPM applied to the public health field—is a dynamic and interactive process of synthesis, dissemination, exchange and ethically sound application of knowledge to improve population health, provide more effective health services and products, and strengthen the health system [3, 4]. Knowledge translation platforms articulate producers, intermediaries and users of scientific knowledge in a complex system of interactions [5], and for their implementation, individual and institutional capacities need to be available and used effectively. These capacities include, for example, implementing structured and replicable research methods, mapping contextual factors that influence a priority public health problem, and choosing, planning, and implementing interventions to address it [6–8].

This set of capacities constitutes a competency profile, including knowledge, skills and attitudes (KSA) integrated for a competent practice, considering cognitive, psychomotor and attitudinal attributes [9–14], which are represented by key actions and typical performances. Key actions delineate activities of a position or function, while performances indicate how the actions should be taken [12, 14]. Thus, competency, based on a constructivist and holistic approach, includes the mobilization of different resources and attributes to address, with relevance and success, complex real-world challenges facing organizations [12, 14].

In the present article, a competency profile essential for practice in EIPM in the Brazilian context was defined. The definition of a set of competency elements required for a professional or social performance is key for identifying capacity gaps in knowledge translation platforms and in organizations working in this field, as well as for defining curricula and structuring courses, favouring the standardization of professional qualifications related to EIPM. In addition, this competency profile contributes to the discussion about the institutionalization of EIPM in Brazil and in the world.

Based on the identification of needs and gaps in the context of institutionalization of EIPM in Brazil, the Brazilian Ministry of Health has commissioned the development of a competency profile for professional practice in EIPM in different areas of the health system. This article aims to describe the processes and outcomes of this competency profile development, which used a constructivist approach and consensus techniques based on previously systematized information, since specific evidence on the Brazilian context was not available. It also addresses how this profile can be used as a tool to support the institutionalization of EIPM, its situational diagnosis, strategic planning, and effective implementation.

## Methods

This study was carried out in accordance with a protocol that included terms of reference developed jointly by the project's coordination team and the Brazilian Ministry of Health. The terms of reference for the constitution of the expert committee, activities and products are available (in Portuguese) in the Additional file 1: Appendix S1.

The steps of the competency profile development consisted of: (1) Constitution of the expert committee, including researchers, professionals with practical experience, managers, and educators; (2) Development of a rapid review on EIPM competency profiles; (3) Identification and delineation of macro problems relating to the scope of the competency profile; and (4) Outlining of general and specific capacities, categorized by key actions.

### Constitution of the expert committee and meetings

The expert committee consisted of 14 experienced professionals in training and practice in EIPM in the Brazilian context: two professionals (KRCA and RBS) appointed by the Ministry of Health; two (RG and SFS) from Hospital Sírio-Libanês—the institution responsible for this study; and 10 Brazilian professionals who are expert in some relevant step for EIPM implementation (DAM, JA, LSB, LP, NMS, PL, SMVLO, SECM, TSD, and TST), selected through purposive sampling by those responsible for this study. The initial sample consisted of 13 experts, evenly distributed by gender and geographical region of Brazil, with the following professional profiles: (1) decision-makers with expertise in EIPM; (2) researchers from universities/research institutes who apply knowledge translation to policy; (3) healthcare workers who use evidence; (4) members of organized civil society or the private sector with expertise in EIPM; and (5) science communication professionals. Three out of the 13 experts declined to participate due to busy schedules. The committee members were invited to contribute as authors of the competency profile, and therefore as part of the research team.

In addition to the experts invited and indicated, the committee included the participation of four facilitators with experience in EIPM and deliberative processes (JOMB, DMMR, CS and MLTM), who were also part of the project's coordination team. This group of facilitators was responsible for: planning, conducting and mediating, recording and producing systematic syntheses and products of the workshops and asynchronous activities; and consolidating the final version of the EIPM competency profile.

The committee attended six online workshops. Each workshop had a specific objective, including defining macro problems, and outlining key actions and performances essential for the EIPM competency profile in the Brazilian context. Individual committee members’ participation in the workshops and asynchronous activities was not anonymous (all participants identified themselves).

A rapid review [14] was used as a starting point for discussion, which synthesized the global reference on the theme, considering that EIPM is a multiprofessional and multidisciplinary field, and a specific regulation for practice in EIPM is not required in Brazil. The rapid review, the macro problems, and the general and specific capacities are detailed below.

### Rapid review on EIPM competency profiles

The rapid review [14] was conducted to address the question: What are the general and specific elements of competency (KSA) for professional training and practice in EIPM? A total of 37 elements of competency were identified, eight categorized as knowledge, 19 as skills, and 10 as attitudes. These elements were aggregated into four competency profiles predetermined by the authors: researcher, health professional and manager, and citizen.

The results of this review were used by the committee members in two ways: (1) an individual prioritization process; and (2) as a starting point for discussion to identify and categorize key actions and performances.

### Outlining the macro problems relating to the scope of the competency profile

Considering macro problems as key elements of a context where changes are required, a competency profile should address them if it is expected to bring significant changes that can represent advances and improvements. Therefore, the guidelines to the development of the EIPM competency profile were defined based on macro problems related to public health policies in Brazil, which were identified previously during the planning phase of this study, and then discussed and validated by the expert committee.

### Outlining general and specific capacities—online workshops and preparation of intermediate documents

Six online workshops (Table 1), using the Zoom Meeting Platform, were held to outline the specific and general key actions and performances that constitute the EIPM competency profile for Brazil. The workshops were held between August and December 2021, in Portuguese, and lasted two hours each; instructional materials were previously sent to support the discussions. They were mediated by three facilitators (JOMB, DMMR and MLTM), and recorded and documented with the assistance of another facilitator (CS). Synthetic reports of the deliberations were prepared and shared with the expert committee members after each workshop, generating a consistent and accessible record for all members.

**Table 1:** Online workshops of the expert committee

Activities	Dates, objectives and description of activities
Workshop 1—presentation and discussion about the project, macro problems, and the competency profile	Date: 8 September 2021 Objective: To present the project, define macro problems, and discuss the findings of the rapid review on EIPM competency profiles [14] Description: The committee discussed and validated: the macro problems to be addressed; the findings of the rapid review on EIPM competency in view of the project objectives; the activities to be developed, and the agreements necessary for the committee’s work
Workshop 2—investigation of the practices of competent professionals	Date: 22 September 2021 Objective: To identify practices of competent professionals Description: Based on the discussions of the workshop 1, the committee members contributed individually, according to their own experiences/opinions, by filling out an online form to prioritize the competency elements (knowledge, skills and attitudes) identified in the rapid review. In the form they could also include suggestions to fill gaps considered essential for the practices of EIPM professionals in Brazil. In the workshop 2, they discussed the results of the form, and systematized the information on competency and performances related to EIPM, from the perspective of professional and social practice
Workshops 3 and 4—development of the competency profile	Dates: 20 October and 8 November 2021 Objective: To develop the competency profile Description: Based on the material produced in the workshops 1 and 2, the committee reviewed and discussed about the performances. Four working groups were constituted to enhance the discussion based on guiding questions. They consolidated and presented the results of the discussions. The facilitators supported the systematization of tables containing: the areas of competency, and the description of the respective key actions and performances
Workshops 5 and 6—conclusion of the competency profile	Dates: 24 November and 13 December 2021 Objective: To conclude the development of the competency profile Description: A first draft of the tables of the competency profile, including key actions and performances, by areas of activity, was previously submitted to the committee for review, and the discussions during the workshops 5 and 6 were mediated by consensus conference. After systematization, the resulting material was reviewed globally and jointly by the committee. All contributions of the committee members were reviewed and incorporated. The product of this construction represents a meta-point of view on the EIPM competency profile for Brazil

After workshop 1, a prioritization process was carried out asynchronously, using the results of the rapid review on EIPM competency profiles [14]. The competency elements were arranged in an online form (Google Forms) and sent via e-mail to the committee members. They individually assigned a value for each competency element, considering an ascending scale of 1 (least important) to 5 (most important), for the four areas of activity in EIPM initially defined: research, management and work in health, and organized civil society. In the online form, it was possible to include suggestions for the writing of the competency elements as well as additional elements. Reminder emails were sent out to ensure that all committee members would contribute to the prioritization.

The results of this survey were systematized by the facilitators (JOMB, DMMR, CS and MLTM), and presented to the committee in the workshop 2. They were instrumental in the discussions that followed, ensuring the efficiency of the consolidation process of the EIPM competency profile.

In the course of the committee’s discussions, the four areas of activity in EIPM initially defined were changed to five related areas: (1) health management; (2) scientific research; (3) knowledge translation; (4) organized civil society; and (5) cross-sectional areas. The description of the boundaries adopted by the committee for each of these areas is presented in the results section of this article. These categories were used by the facilitators (JOMB, DMMR and MLTM) in the systematization of consolidated tables of the competency profile, at each stage of development, considering the results of the initial prioritization as well as the contributions from the committee during the online workshops and the asynchronous activities.

The online workshops were supported by preparatory materials distributed by email in advance, and conducted following procedures designed to promote equitable and effective participation by the committee members, alternating between discussions in four small groups of members from different sectors, and plenaries with the full committee. The small group discussions focused on outlining and writing the key actions and related performances for the different areas that would constitute the EIPM competency profile. The plenary focused on providing inputs to the discussions, analysing the results of the small group discussions and the state of the art of the competency profile consolidated in each workshop. All discussions were mediated by the group of facilitators in accordance with a planning that was shared in advance. Table 1 provides a summary of the online workshops of the committee.

## Results

The online workshops were held according to a prior planning. There was an average attendance of at least 80% of the expert committee members at each meeting, and all members attended at least four workshops and contributed to the asynchronous activities (e.g., prioritization of the competency elements). There were no withdrawals, or losses for other reasons, in the course of the expert committee’s discussions, taking into consideration the average individual attendance at the online workshops and asynchronous activities.

The expert committee validated the following macro problems in the context of EIPM in Brazil: (1) lack of systematic and transparent decision-making processes in health policy management; (2) underdeveloped institutional capacity for knowledge management and translation; and (3) incipient use of scientific evidence in the formulation and implementation of health policies. The deliberations on the elements of the EIPM competency profile were based on these macro problems.

### Categorization of the EIPM competency profile

The different key actions and performances of the EIPM competency profile were grouped into the prevalent areas of activity. The areas of activity in EIPM were defined by the expert committee as presented in Table 2.

**Table 2:** Areas of activity in evidence-informed policymaking (EIPM)

Area	Description
Health management	Adopted when the key actions or performances were primarily related to decision-making in health policies, systems and services, including the decision-making itself and the support for its realization
Scientific research	Adopted when the key actions or performances were primarily related to the production of scientific research, at the institutional level and focused on the production of valid knowledge that can be used to improve health policies and systems
Knowledge translation	Adopted when the key actions or performances were primarily related to activities for the implementation and development of knowledge translation platforms, aimed at the synthesis, dissemination, exchange and ethically sound application of knowledge in health policies and systems
Organized civil society	Adopted when the key actions or performances were primarily related to participation in organized social movements
Cross-sectional	Adopted when it was considered that the key actions or performances were related in an integrated way to all areas of activity (management, research, knowledge translation, and organized civil society)

Subsequently, the key actions and performances were also categorized according to their level of coverage in relation to EIPM: ‘specific’ when these elements would be essential for the development of activities specific to EIPM; and ‘general’ when they would be related to EIPM but also integrating a broader set of knowledge, skills and attitudes for competent practice, according to each area. An ‘area’ was considered an environment including different themes and correlated or similar dynamics. For example, the key action ‘Use evidence’ is classified as specific, because it is typical of work in EIPM. The key action ‘Know the fundamentals of scientific research’ is classified as general, since it is relevant to EIPM, but it also constitutes the profile of several positions and functions.

Furthermore, a classification based on the KSA acronym (for Knowledge, Skills and Attitudes) was applied to the key actions and performances, considering the following definitions [15]:

- Knowledge: a set of information, facts, theories, practices, and principles necessary to exercise an occupation or to obtain a professional qualification.
- Skills: ability to apply knowledge and use acquired resources to complete tasks and solve problems. It can be cognitive, practical, physical, psychomotor, or sensory.
- Attitudes: ability to develop tasks and solve problems with varying degrees of autonomy and responsibility. These are individual attributes that can influence performance at work. They are organized into four categories: work under supervision; autonomy in one’s own work; supervision of others' work; and evaluation of work or activity.

The distinction between knowledge, skills and attitudes was not always seen as clear or unambiguous by the expert committee. It was understood that performances imply, at different levels, knowledge, skills and attitudes. Therefore, the classification indicated the elements predominant in each performance, i.e., a performance classified as a skill could also include related knowledge and attitudes, and vice versa. The notation adopted in the classification of the key actions and performances based on KSA used capital letters (‘K’ for Knowledge, ‘S’ for Skills, and ‘A’ for Attitudes), in the competency profile table.

### Key actions and performances of the EIPM competency profile

A total of 42 key actions relevant to the practice in EIPM were identified, defined, and distributed in five areas of activity (Table 3), and their attributes and respective performances are detailed in the EIPM competency profile (Table 4).

**Table 3:** Summary table of key actions for evidence-informed policymaking (EIPM) according to areas of activity

Key action	Health Management	Scientific research	Civil society	Knowledge translation
Supporting the institutionalization of the use of evidence	X			
Improving evidence communication	X	X	X	X
Acting with confidence in one’s own abilities	X	X	X	X
Acting with motivation and initiative	X	X	X	X
Acting with professionalism and ethics	X	X	X	X
Acting for the benefit of citizens	X	X	X	X
Evaluating health policies	X			
Combining different types of evidence				X
Understanding health policies	X			
Communicating and disseminating evidence	X	X	X	X
Knowing the organizational context	X			
Knowing the fundamentals of scientific research		X		
Knowing health systems	X			
Contextualizing evidence	X			
Establishing good interpersonal relationships for collaborative processes	X	X	X	X
Trusting the other partners and actors in the system	X	X	X	X
Extracting scientific information		X		
Facilitating group interaction	X	X	X	X
Advocating for EIPM			X	
Formulating EIPM	X			
Managing knowledge translation actions				X
Managing conflicts of interest	X			
Managing people and teams	X			
Managing organizational processes	X			
Managing projects	X			
Implementing EIPM	X			
Leading knowledge translation processes and projects	X	X	X	X
Having basic computer skills	X	X	X	X
Mobilizing collaborative networks	X			
Prioritizing questions and problems				X
Promoting cooperative actions for EIPM	X	X	X	X
Conducting scientific research		X		
Conducting knowledge translation				X
Reflecting carefully	X	X	X	X
Appreciating teamwork	X	X	X	X
Using evidence	X			
Valuing learning	X	X	X	X
Valuing creativity	X	X	X	X
Valuing social participation	X	X	X	X
Valuing scientific research	X	X	X	X
Valuing transparency in EIPM	X	X	X	X
Appreciating the possibility of change	X	X	X	X

**Table 4:** Evidence-informed policymaking (EIPM) competency profile

Key action	Performance description	
Area of activity—health management		
Specific		
Supporting the institutionalization of the use of evidence	To support and publicly promote the use of scientific evidence in the institution, through processes and mechanisms of knowledge translation in all its stages (synthesis, dissemination, exchange and ethically sound application).	**A﻿**
Knowing the organizational context	To know the structure, dynamics and capacities to formulate and implement EIPM in relevant organizations, according to their contexts.	**K**
Contextualizing evidence	To use scientific evidence in a contextualized way, identifying and implementing adaptations when necessary.	**S**
Using evidence	To use the best available evidence to identify, in a systematic and transparent manner, potential benefits, risks, uncertainties, and costs arising from the decisions.	
Formulating EIPM	To plan and formulate evidence-informed health programs and policies in a systematic, transparent and participatory way.	
Implementing EIPM	To implement and monitor health programs and policies by developing and adopting contextualized and evidence-informed implementation plans.	
Evaluating health policies	To evaluate public health policy implementation processes and their outcomes (e.g., products, processes, impacts).	
Mobilizing collaborative networks	To mobilize collaborative networks among EIPM stakeholders, respecting institutional and cultural norms and practices.	
General		
Understanding health policies	To understand what public health policies are, and the processes involved in their development and implementation.	**K**
Knowing health systems	To know the structure and dynamics of health systems and their organizations, in their different spheres of management and levels of care.	
Managing organizational processes	To manage organizational processes in health system institutions at their specific level of practice.	**S**
Managing projects	To manage resources, processes and risks, and carry out monitoring and evaluation of projects related to health systems and policies.	
Managing people and teams	To coordinate, mobilize, and engage individuals and teams to achieve the institutional objectives.	
Managing conflicts of interest	To manage conflicts of interest, minimizing competition and maximizing collaboration.	
Area of activity—scientific research		
Specific		
Conducting scientific research	To produce, search, critically assess and synthesize scientific evidence.	**S**
General		
Knowing the fundamentals of scientific research	To know methods, techniques and resources of scientific research.	**K**
Extracting scientific information	To extract information from scientific texts relevant to a certain field, written in languages other than the native one.	**S**
Area of activity—civil society		
Specific		
Advocating for EIPM	To advocate or promote EIPM with civil society, governments and decision-makers, research groups and institutions.	**S**
Area of activity—knowledge translation		
Specific		
Prioritizing questions and problems	To identify and prioritize questions and problems relevant to the context of health policies and systems.	**S**
Managing knowledge translation actions	To plan and apply organizational knowledge translation strategies in the context of practice.	
Conducting knowledge translation	To apply knowledge translation methods, mechanisms and processes.	
Combining different types of evidence	To combine scientific evidence with other types of evidence to inform the decision-making process in health systems and policies.	
Area of activity—cross-sectional		
Specific		
Valuing scientific research	To value scientific research as an important resource in decision-making in health policies and systems, at all its stages and levels.	**A**
Trusting the other partners and actors in the system	To establish and maintain relationships of mutual trust based on transparency with the actors involved in the EIPM processes in their own environments and contexts.	
Valuing transparency in EIPM	To recognize, advocate and adopt transparency as a principle of action, so that EIPM processes are reliable and accessible to all stakeholders.	
Communicating and disseminating evidence	To communicate and disseminate scientific evidence to different audiences, publicly promoting its social appropriation.	**S**
Improving evidence communication	To continuously enhance the use of evidence communication techniques and resources in the context of health policy and systems.	
Leading knowledge translation processes and projects	To lead knowledge translation processes and projects, promoting the engagement of the responsible team and relevant key actors.	
Promoting cooperative actions for EIPM	To establish and encourage the creation of bonds, partnerships and exchanges for cooperation and teamwork among decision-makers, researchers, and other actors involved in EIPM.	
Having basic computer skills	To properly manage computer resources (software and hardware) and other information technologies important to the practice and development of EIPM.	
General		
Acting for the benefit of citizens	To work for a broad conception of the right to health, advocate and prioritize the quality and sustainability of health policy, systems and services.	**A**
Reflecting carefully	To carefully, judiciously and sensibly reflect on problems and dilemmas related to public health policies, with a balanced judgment.	
Acting with professionalism and ethics	To act with high ethical and professional standards, guided by principles of integrity, responsibility, commitment to learning, and continuous improvement of practice.	
Valuing social participation	To recognize, advocate and act to value participatory processes in health policy and systems.	
Appreciating teamwork	To adopt practices that promote collaborative teamwork.	
Valuing learning	To have a lifelong commitment to self-directed learning based on critical and reflective thinking.	
Valuing creativity	To value creativity to solve problems, combining strategies and resources in a proactive and participatory manner.	
Appreciating the possibility of change	To have a flexible personal and professional attitude, accepting, valuing, enabling and managing the occurrence of situations that bring change.	
Acting with confidence in one’s own abilities	To make an assertive use of one’s already developed knowledge, skills and attitudes.	
Acting with motivation and initiative	To act with motivation and initiative, proactively seeking opportunities to contribute to improving the environment and context of practice.	
Facilitating group interaction	To apply techniques for facilitating groups, aimed at exchange and cooperation in the collective construction of knowledge and practices in health.	**S**
Establishing good interpersonal relationships for collaborative processes	To establish good interpersonal relationships based on ethical and respectful practices for collaboration, regardless of the hierarchical position occupied.	

*A* Attitude, *K* Knowledge, *S* Skill

## Discussion

This article described an evidence-informed policymaking (EIPM) competency profile for Brazil that can be considered for application in different local and national contexts. The elements of competency essential for EIPM consist of an integrated and interactive set of individual capacities that interacts with the organizational environment, constituting a professional profile with different areas of activity.

The competency profile is a tool to support the diagnosis and planning of actions for the institutionalization of EIPM, and the effective incorporation of scientific evidence into decision-making in health policies and systems. The participatory process reported in this study was aimed at identifying the essential elements for a EIPM competency profile in Brazil.

Many studies address theoretical and operational elements of competency related to EIPM [16–21], but the contextualization process conducted by the expert committee made it possible to re-signify these elements for the Brazilian national context, since all the process strongly considered the experience and knowledge of the committee participants. This contextualization of the process can also be considered in perspective, to confront and interpret the results presented here in the face of other initiatives for categorization of competence elements for different contexts. In this respect, the competence profile presented here is similar to the set of scientific publications that addressed this topic, with the advantage of providing a comprehensive view and covering different profiles that work in EIDM.

At the same time, synthetic definitions applicable to institutional processes at different levels of organizational complexity were formulated. The practical application of the competency profile developed should consider the local needs of each individual or institution. The use of this tool to support the advancement of EIPM institutionalization should be based on situational diagnosis and strategic planning focused on enhancing the use of evidence in health policies and systems, so that the contextualization process can enable the adaptation of the competency profile to each situation and context.

In the scope of EIPM, the importance of strengthening its institutionalization within governments, civil society organizations, and academic institutions is recognized worldwide [22–24], but many barriers still need to be addressed in a structured way [25]. It is expected that with the competency profile presented here the EIPM ecosystem will be better equipped to identify the elements of competency that need to be developed in these institutionalization processes.

### Application of the EIPM competency profile

In the course of the expert committee’s discussions, it became clear that this competency profile should not be interpreted as the profile of a single professional. The range of performances described in this study constitutes a set of knowledge, skills and attitudes necessary for competent institutional practice in EIPM. In other words, it is not reasonable to expect that a single person possesses all the attributes described above, but that a group of professionals, working as a team or in partnership, can have the necessary profile.

Similarly, this competency profile does not relate to a specific institution or area of activity, but it is aimed at organizing performances relevant to the practice in EIPM in various contexts. Therefore, the first step in applying this profile should be to analyse which key actions are relevant to the concrete application context so as to have a customized profile. For example, a health department may not need to include the key action ‘Advocating for EIPM’ in its competency profile, while a university may not include the key action ‘Implementing EIPM’.

The selection of the key actions relevant to each context can also be accompanied by a second classification in terms of ‘depth’, ‘frequency’, and ‘importance’. This classification makes it possible to prioritize performances according to different levels of occupation. Thus, even though ‘Conducting scientific research’ may be a key action to both a health manager and a researcher, the frequency and importance of this skill will be different for both.

Therefore, this competency profile should be used as an initial matrix for defining specific profiles. These profiles can modulate, prioritize and change the general profile, adding or removing performances, in order to make it suitable for the concrete organizational needs. Based on these adaptations, it will be possible to design a profile for specific positions and functions, as well as competency profiles for training activities.

## Conclusions

This article presented the process and outcomes of the development of a EIPM competency profile for Brazil. These outcomes have the potential to contribute to the institutionalization of the systematic and transparent use of scientific evidence to inform decision-making in health policies and systems. The competency profile presented here delineate performances and key actions, in different areas, to advance EIPM in Brazil.

Finally, taking as a reference the advancement of EIPM in Brazil and in other parts of the world, aspects or parts of the competency profile can contribute to the development of curricula for courses at different levels, selection of professionals, evaluation of professional performance, career advancement plan, and guidelines for the formation of learning communities in institutional environments.

### Supplementary Information

**Additional file 1: Appendix S1** Terms of reference for the expert committee and workshops (only in Portuguese)

## Data Availability

Available in Additional file 1: Appendix S1.

## Abbreviations

EIDM: Evidence-informed decision making
EIPM: Evidence-informed policymaking
ESPIE: Project “Support for the Formulation and Implementation of Evidence-Informed Public Health Policy”
KSA: Knowledge, skills and attitudes
