# Supplementary material for: An evidence-informed policymaking (EIPM) competency profile for the Brazilian Health System developed through consensus: process and outcomes
Source: Health Res Policy Syst. 2023 Oct 12;21:105. doi: 10.1186/s12961-023-01052-z (PMC10571264; doi:10.1186/s12961-023-01052-z)
Supplement: Supplementary file 1 — Additional file 1: Appendix S1 Terms of reference for the expert committee and workshops (only in Portuguese) [file 12961_2023_1052_MOESM1_ESM.pdf]

Apoio à Formulação e Implementação de Políticas de Saúde Informadas por Evidências – ESPIE

2021-2023

HOSPITAL SÍRIO-LIBANÊS · CONASEMS · CONASS · PROADI-SUS · SUS · MINISTÉRIO DA SAÚDE · PÁTRIA AMADA BRASIL GOVERNO FEDERAL

Perfil de competência do profissional em PIE no Brasil

Grupo de autoria

Termos de referência das oficinas de trabalho

Setembro / 2021

Prezado(a) participante do Grupo de Autoria,

Agradecemos por você ter aceitado o nosso convite para integrar o Grupo de Autoria do perfil de competência do profissional de PIE, no âmbito do projeto '**ESPIE - Apoio à formulação e implementação de políticas de saúde informadas por evidências**'.

O projeto ESPIE é desenvolvido pelo Hospital Sírio-Libanês (HSL), como parte do PROADI-SUS. Seu objetivo geral é prestar apoio técnico, científico e institucional para a promoção do uso sistemático e transparente das evidências científicas na tomada de decisão em saúde e para a implementação de Políticas Informadas por Evidências (PIE) no SUS.

No contexto deste projeto, será desenvolvido o **perfil de competência do profissional de PIE** para o Brasil, o qual subsidiará atividades a serem implementadas pelo HSL, em especial aquelas com objetivos educacionais. Consideramos que sua experiência e conhecimento profissionais serão relevantes para como parte do grupo de autoria que desenvolverá o perfil de competência acima mencionado.

Sua participação no grupo de autoria será voluntária e reconhecida, e implicará no seu compromisso de leitura de materiais informativos e de participação em cinco oficinas *online* (via ZOOM), com até 3 horas de duração, com os temas e datas e horários:

Oficina 1 – Apresentação e discussão sobre o projeto ESPIE, o perfil de competência e a Revisão Crítica Rápida	08/09/21
Oficina 2 - Investigação das práticas profissionais competentes	22/09/21
Oficina 3 - Elaboração do perfil profissional – parte 1	20/10/21
Oficina 4 - Elaboração do perfil profissional – parte 2	10/11/21
Oficina 5 - Validação do perfil profissional	24/11/21

A seguir, você encontrará os termos de referência para estas oficinas, incluindo os objetivos e os programas de cada uma delas.

Juntamente com este documento, você também está recebendo, para leitura prévia, o relatório da revisão crítica rápida 'Perfis de competência para políticas informadas por evidências (PIE)', bem como a nota técnica do HSL sobre elaboração de perfis de competência e o quadro informativo do perfil de competência de PIE, desenvolvido na edição anterior do projeto ESPIE. Estes documentos serão usados como 'disparadores' da discussão, a partir da primeira oficina.

Perfil de competência do profissional em PIE no Brasil (Entrega 4)

Termo de Referência

Oficina 1 - Apresentação e discussão sobre o projeto ESPIE, o perfil de competência e a Revisão Crítica Rápida

Facilitadores: Jorge Barreto e Davi Romão

Data/Horário: 08/09/21, 9h-12h00

Objetivos: Apresentar o projeto ESPIE e discutir os achados da revisão crítica rápida sobre competências para PIE

Descrição: Atores relevantes na regulação, formação, atuação ou regulamentação da função em questão compõem o grupo de autoria e representam distintos pontos de vista da sociedade em relação ao que considera como prática competente do profissional de PIE. Estes atores serão apresentados ao projeto e suas intencionalidades, com foco na construção do perfil de competência em PIE para o Brasil. A oficina tem a intencionalidade de discutir os achados da revisão crítica rápida sobre competências para PIE em face dos objetivos estruturados do projeto ESPIE. Também serão apresentadas as atividades a serem desenvolvidas e obtidos os acordos necessários ao trabalho do grupo de autoria.

Documentos:

- TR da oficina 1
- Relatório da revisão crítica rápida sobre competências em PIE
- Nota técnica do IEP-HSL sobre Perfil de Competência
- Perfil de competência do ESPIE (edição anterior, para formação de especialistas em PIE)

Programa:

9h – 9h30: Apresentação dos participantes (todos, 10 min), apresentação do projeto ESPIE (Sílvio Fernandes, 5 min), seguida de discussão sobre o projeto ESPIE (todos, 15 min) (Facilitador: Jorge Barreto)

9h30 – 10h: Exposição dialogada sobre a construção do perfil de competência e sobre os objetivos, atividades / dinâmica do grupo de autoria e produto esperado (Jorge Barreto, 10 min), seguida de discussão sobre os elementos apresentados (todos, 20 min) (Facilitador: Jorge Barreto)

10h – 10h30: Discussão sobre os acordos necessários para o trabalho do grupo de autoria (todos, 30 min) (Facilitador: Jorge Barreto)

10h30 – 10h45: Intervalo

10h45 – 11h05: Apresentação achados RCR (20 min) (Facilitadores: Davi Romão / Cecilia Setti)

11h05 – 11h40: Discussão sobre RCR (35 min) (Facilitadores: Davi Romão / Jorge Barreto)

11h40 – 12h00: Finalização e encaminhamentos (20 min) (Facilitadores: Davi Romão / Jorge Barreto)

Perfil de competência do profissional em PIE no Brasil (Entrega 4)

Termo de Referência

Oficina 2 - Investigação das práticas de profissionais competentes

Facilitadores: Jorge Barreto e Davi Romão

Data/Horário: 22/09/21, 9h-12h00

Objetivos: Investigar as práticas de profissionais competentes

Descrição: A partir das discussões realizadas na oficina 1, o grupo de autoria será previamente convidado a contribuir individualmente, segundo suas próprias experiências/opiniões, com o preenchimento de formulário online de priorização dos elementos de competência (conhecimentos, habilidades e atitudes) identificados na revisão rápida. O formulário também possibilitará o envio de sugestões para suprir lacunas que os membros do grupo de autoria considerem essenciais às práticas dos profissionais de PIE no Brasil. A oficina terá a intencionalidade de discutir e sistematizar informações sobre competências e desempenhos relacionados a PIE, na perspectiva das práticas profissionais, a partir dos subsídios trazidos individualmente pelos membros do grupo de autoria. Essa discussão também será articulada com o quadro de referência do projeto e com os resultados da discussão sobre os achados da revisão crítica rápida sobre competências para PIE.

Documentos:

- TR da oficina 2
- Lista de participantes
- Informativo sobre a plataforma online
- Reporte breve da oficina anterior
- Sistematização das respostas ao formulário online de priorização dos elementos de competência (conhecimentos, habilidades e atitudes)

Programa:

9h – 9h10: Abertura da oficina. Tira-dúvidas sobre o programa (todos, 10 min) (Facilitador: Jorge Barreto)

9h10 – 9h30: Apresentação dos resultados da sistematização das respostas ao formulário online de priorização, segundo elementos de competência (conhecimentos, habilidades e atitudes) e perfis profissionais (Facilitador: Davi Romão, 20 min)

9h30 – 10h30: Discussão dos resultados apresentados (todos, 60 min) (Facilitadores: Jorge Barreto / Davi Romão)

10h30 – 10h45: Intervalo

10h45 – 11h45: Continuação - Discussão dos resultados apresentados (todos, 60 min) (Facilitadores: Jorge Barreto / Davi Romão)

11h45 – 12h00: Finalização e encaminhamentos (todos, 15 min) (Facilitadores: Jorge Barreto / Davi Romão)

Perfil de competência do profissional em PIE no Brasil (Entrega 4)

Termo de Referência

Oficina 3 - Elaboração do perfil profissional – parte 1

Facilitadores: Jorge Barreto e Davi Romão

Data/Horário: 20/10/21, 9h-12h00

Objetivos: Elaborar o perfil profissional de PIE

Descrição: A partir do material produzido nas oficinas de trabalho anteriores (elementos de competência considerados essenciais), o grupo de autoria analisará e discutirá os subsídios para a elaboração do texto descritivo para cada desempenho conexo. Os facilitadores, a partir desses materiais, apoiarão a elaboração de quadros contendo: áreas de competência, descrição das suas respectivas ações-chave e desempenhos. Uma adaptação da técnica de análise funcional, somada à análise do trabalho em sua dinâmica, deve ser utilizada para a produção dos quadros que descrevem as áreas de competência, ações-chave e desempenhos.

Documentos:

- TR da oficina 3
- Lista de participantes
- Informativo sobre a plataforma online
- Reporte breve da oficina anterior
- Quadros de competência (conhecimentos, habilidades e atitudes), segundo perfis profissionais

Atividades:

9h – 9h10: Abertura da oficina. Tira-dúvidas sobre o programa (todos, 10 min) (Facilitador: Jorge Barreto)

9h10 – 9h30: Apresentação da síntese das discussões da oficina 2 (Davi Romão, 10 min), seguida de discussão (todos, 20 min) (Facilitador: Jorge Barreto)

9h30 – 10h30: Discussão sobre a elaboração do texto descritivo para cada desempenho conexo aos elementos de competência considerados essenciais, segundo perfil profissional. Concomitantemente, criação dos quadros contendo: áreas de competência, descrição das suas respectivas ações-chave e desempenhos (todos, 60 min) (Facilitadores: Jorge Barreto / Davi Romão)

10h30 – 10h45: Intervalo

10h45 – 11h45: Continuação - Discussão sobre a elaboração do texto descritivo para cada desempenho conexo aos elementos de competência considerados essenciais, segundo perfil profissional. Concomitantemente, criação dos quadros contendo: áreas de competência, descrição das suas respectivas ações-chave e desempenhos (todos, 60 min) (Facilitadores: Jorge Barreto / Davi Romão)

11h45 – 12h00: Finalização e encaminhamentos (todos, 15 min) (Facilitadores: Jorge Barreto / Davi Romão)

Perfil de competência do profissional em PIE no Brasil (Entrega 4)

Termo de Referência

Oficina 4 - Elaboração do perfil profissional – parte 2

Facilitadores: Jorge Barreto e Davi Romão

Data/Horário: 10/11/21, 9h-12h00

Objetivos: Elaborar o perfil profissional de PIE (continuação)

Descrição: Continuidade da oficina 3, a partir do material produzido nas oficinas de trabalho anteriores (elementos de competência considerados prioritários e essenciais), o grupo de autoria analisará e discutirá os subsídios para a elaboração do texto que descreve cada desempenho conexo. Os facilitadores, a partir desses materiais, devem elaborar os quadros contendo as áreas de competência e suas respectivas ações-chave e desempenhos. Uma adaptação da técnica de análise funcional, somada à análise do trabalho em sua dinâmica, deve ser utilizada para a produção dos quadros que detalham as áreas de competência, segundo ações-chave e desempenhos.

Documentos:

- TR da oficina 4
- Lista de participantes
- Informativo sobre a plataforma online
- Reporte breve da oficina anterior
- Quadros de competência (conhecimentos, habilidades e atitudes) e desempenhos, segundo perfis profissionais

Atividades:

9h – 9h10: Abertura da oficina. Tira-dúvidas sobre o programa (todos, 10 min) (Facilitador: Jorge Barreto)

9h10 – 9h30: Apresentação da síntese das discussões da oficina 3 (Davi Romão, 10 min), seguida de discussão (todos, 20 min) (Facilitador: Jorge Barreto)

9h30 – 10h30: Discussão sobre a elaboração do texto descritivo para cada desempenho conexo aos elementos de competência considerados essenciais, segundo perfil profissional. Concomitantemente, criação dos quadros contendo: áreas de competência, descrição das suas respectivas ações-chave e desempenhos (todos, 60 min) (Facilitadores: Jorge Barreto / Davi Romão)

10h30 – 10h45: Intervalo

10h45 – 11h45: Continuação - Discussão sobre a elaboração do texto descritivo para cada desempenho conexo aos elementos de competência considerados essenciais, segundo perfil profissional. Concomitantemente, criação dos quadros contendo: áreas de competência, descrição das suas respectivas ações-chave e desempenhos (todos, 60 min) (Facilitadores: Jorge Barreto / Davi Romão)

11h45 – 12h00: Finalização e encaminhamentos (todos, 15 min) (Facilitadores: Jorge Barreto / Davi Romão)

Perfil de competência do profissional em PIE no Brasil (Entrega 4)

Termo de Referência

Oficina 5 - Validação do perfil profissional

Facilitadores: Jorge Barreto e Davi Romão

Data/Horário: 24/11/21, 9h-12h00

Objetivos: Validar o perfil de competência em PIE para o Brasil

Descrição: A primeira versão dos quadros de competência, segundo perfil profissional, incluindo os elementos de competência, ações-chave e desempenhos, será submetida previamente à validação do grupo de autoria, por meio de abordagem de conferência de consenso. Após a validação, o material resultante será avaliado de forma global e conjunta pelo grupo de autoria. Todas as contribuições da validação serão analisadas. O produto dessa construção deve representar um meta-ponto de vista sobre o perfil de competência do profissional de PIE para o Brasil.

Documentos:

- TR da oficina 4
- Lista de participantes
- Informativo sobre a plataforma online
- Reporte breve da oficina anterior
- Informativo sobre a ferramenta de validação individual
- Resultados da validação (Delphi)
- Primeira versão do perfil de competência

Atividades:

9h – 9h10: Abertura da oficina. Tira-dúvidas sobre o programa (todos, 10 min) (Facilitador: Jorge Barreto)

9h10 – 9h30: Apresentação da síntese das discussões da oficina 4 (Davi Romão, 10 min), seguida de discussão (todos, 20 min) (Facilitador: Jorge Barreto)

9h30 – 10h30: Apresentação e discussão dos quadros de competência, segundo perfil profissional, após a validação (todos, 60 min) (Facilitadores: Jorge Barreto / Davi Romão)

10h30 – 10h45: Intervalo

10h45 – 11h15: Continuação - Apresentação e discussão dos quadros de competência, segundo perfil profissional, após a validação (todos, 30 min) (Facilitadores: Jorge Barreto / Davi Romão)

11h15 – 12h00: Finalização e encaminhamentos (todos, 45 min) (Facilitadores: Jorge Barreto / Davi Romão)

Participantes

n	Nome	E-mail	Instituição	Setor
1	Daienne Machado	daienne.machado@codeplan.df.gov.br	CODEPLAN/DF	Gestão
2	Sara Mota	sara.mota@saude.gov.br	Superintendência MS-BA	Gestão
3	Tamille Dias	tamille.dias@enap.gov.br	ENAP	Gestão
4	Tereza Toma	ttoma.ats@gmail.com	Instituto de Saúde/SP	Pesquisa
5	Sandra Leone	sandrinhaleone@gmail.com	UFMS	Pesquisa
6	Nathan M Sousa	nathanmendes@hotmail.com	Médico /UFMG	Serviço
7	João Abreu	joao@impulsogov.org	Impulso.gov	Sociedade
8	Laura Boeira	laura@veredas.org	Instituto Veredas	Sociedade
9	Patrícia Logullo	patricia.logullo@ndorms.ox.ac.uk	Equator/Oxford	Comunicação
10	Letícia Pozza	leticia@odd.group	Ods Studio	Comunicação
11	Silvio Fernandes	fernandessilvio90@gmail.com	IEP-HSL	Pesquisa
12	Romeu Gomes	romeu.gomes@hsl.org.br	IEP-HSL	Pesquisa
13	Roberta Borges Silva	roberta.silva@saude.gov.br	DECIT/SCTIE/MS	Gestão
14	Keitty Andrade	keitty.andrade@saude.gov.br	DECIT/SCTIE/MS	Gestão

Facilitadores

n	Nome	e-mail	Instituição	Setor
1	Jorge Barreto	jorgeomaia@hotmail.com	IEP-HSL/Fiocruz	Pesquisa
2	Davi Mamblona	davi@veredas.org	IEP-HSL/Veredas	Pesquisa
3	Maria Lúcia Machado	mluciatmachado@gmail.com	IEP-HSL	Pesquisa

HOSPITAL SÍRIO-LIBANÊS CONASEMS CONASS PROADI-SUS SUS MINISTÉRIO DA SAÚDE PÁTRIA AMADA BRASIL GOVERNO FEDERAL

HOSPITAL
SÍRIO·LIBANÊS

SOCIEDADE BENEFICENTE DE SENHORAS

hsl.org.br
